# Serum biomarker panels for the diagnosis of gastric adenocarcinoma

**DOI:** 10.1038/bjc.2011.592

**Published:** 2012-01-12

**Authors:** H S Ahn, Y S Shin, P J Park, K N Kang, Y Kim, H-J Lee, H-K Yang, C W Kim

**Affiliations:** 1 Department of Surgery, Seoul National University-Boramae hospital, Seoul, Korea; 2 BioInfra Inc., Cancer Research Institute, Seoul National University College of Medicine, Seoul, Korea; 3 Department of Statistics, Seoul National University College of Natural Sciences, Seoul, Korea; 4 Department of Surgery and Cancer Research Institute, Seoul National University College of Medicine, Seoul, Korea; 5 Department of Pathology, Cancer Research Institute, and Tumor Immunity Medical Research Center, Seoul National University College of Medicine, Seoul, Korea

**Keywords:** biomarker, gastric adenocarcinoma, diagnosis, screening

## Abstract

**Background:**

Currently, serum biomarkers, which are sufficiently sensitive and specific for early detection and risk classification of gastric adenocarcinoma do not exist. Therefore, this study identified a panel of serum biomarkers for the diagnosis of gastric adenocarcinoma.

**Methods:**

A 29-plex array platform with 29 biomarkers, consisting of 11 proteins discovered through proteomics and 18 previously known to be cancer-associated, was constructed. A test/training set consisting of 120 gastric adenocarcinoma and 120 control samples were examined. After 13 proteins were selected as candidate biomarkers, multivariate classification analyses were used to identify algorithms for diagnostic biomarker combinations. These algorithms were independently validated using a set of 95 gastric adenocarcinoma and 51 control samples.

**Results:**

Epidermal growth factor receptor (EGFR), pro-apolipoprotein A1 (proApoA1), apolipoprotein A1, transthyretin (TTR), regulated upon activation, normally T-expressed and presumably secreted (RANTES), D-dimer, vitronectin (VN), interleukin-6, α-2 macroglobulin, C-reactive protein and plasminogen activator inhibitor-1 were selected as classifiers in the two algorithms. These algorithms differentiated between the majority of gastric adenocarcinoma and control serum samples in the training/test set with high accuracy (>88%). These algorithms also accurately classified in the validation set (>85%).

**Conclusion:**

Two panels of combinatorial biomarkers, including EGFR, TTR, RANTES, and VN, are developed, which are less invasive method for the diagnosis of gastric adenocarcinoma. They could supplement clinical gastroscopic evaluation of symptomatic patients to enhance diagnostic accuracy.

Gastric adenocarcinoma is the most common malignancy in Korea, and was the second leading cause of death by malignancy (19.4%) in 2001 (Lee *et al*, 2002; Ministry of Health and Welfare, 2003). Worldwide, it is the fourth most common cancer and the second most common cause of cancer-related deaths (700 000 deaths per year) following lung cancer (1.18 million deaths per year) (Parkin *et al*, 2005). Survival rates for gastric cancer patients are considerably lower than that of other common cancers, except for cancers of the liver, pancreas, and oesophagus. Five-year survival rates of patients with localised disease (61%) decreases to 25% after the cancer spreads to regional lymph nodes, and to 4% following distant metastasis (Jemal *et al*, 2008). Patients with symptomatic gastric cancer typically have more advanced lesions and shorter survival rates than asymptomatic patients (Kong *et al*, 2004). Early detection and proper treatment following precise risk classification are crucial for improving the outcome of gastric adenocarcinoma. Gastroscopic examination is the most reliable method for diagnosis of gastric adenocarcinoma. The high incidence of gastric adenocarcinoma led to the broad practice of gastroscopy in Korea and Japan. However, the low incidence in most countries resulted in positive predictive values of only 0.4–0.7 for this invasive approach. Therefore, the feasibility and effectiveness of gastroscopy is questionable for these countries (Maniatis *et al*, 1997; Bustamante *et al*, 2002; Genta, 2004).

Biomarkers that identify patients at high risk for gastric adenocarcinoma would increase the predictive value of endoscopy and have clinical benefits for detecting gastric adenocarcinoma. Therefore, extensive research has revealed several serum biomarkers for gastric cancer, including carcinoembryonic antigen (Reiter *et al*, 1997; Marrelli *et al*, 1999, 2001; Gaspar *et al*, 2001; Ishigami *et al*, 2001), cancer antigen 19-9 (Reiter *et al*, 1997; Marrelli *et al*, 1999, 2001; Gaspar *et al*, 2001; Ishigami *et al*, 2001), cancer antigen 72-4 (Kodama *et al*, 1995; Reiter *et al*, 1997; Marrelli *et al*, 1999, 2001; Gaspar *et al*, 2001; Ishigami *et al*, 2001), E-cadherin (Juhasz *et al*, 2003), pepsinogen (Miki *et al*, 2003), cytokines, and cytokeratin fragments (Wu *et al*, 1996; Nakata *et al*, 1998; Forones *et al*, 2001; Ikeguchi *et al*, 2009; Kim *et al*, 2009b). However, the sensitivity of the previously identified serum biomarkers was not sufficient for diagnosis of gastric adenocarcinoma because the sensitivity of tumour markers such as carcinoembryonic antigen, cancer antigen19-9, and CA72-4 was low (20–30%) (Kodama *et al*, 1995; Reiter *et al*, 1997; Marrelli *et al*, 1999, 2001; Gaspar *et al*, 2001; Ishigami *et al*, 2001).

In this study, we developed diagnostic biomarker panel algorithms and validated their performance differentiating patients with gastric adenocarcinoma from controls.

## Patients and methods

### Patient samples

Serum samples were collected from patients with newly diagnosed primary gastric adenocarcinoma without the presence of other cancers at Seoul National University Hospital. Ninety-two serum samples were obtained between November 2002 and December 2003 (period 1), and 123 serum samples were obtained between July 2006 and August 2007 (period 2). Control samples were collected from attendees of the cancer-screening programme of the Seoul National University Healthcare System between January and December 2004. All participants underwent (1) history taking, (2) physical examination, (3) routine blood and *H. pylori* IgG tests, (4) chest X-ray, (5) abdominal sonography or computed tomography, (6) esophagogastroduodenoscopy, (7) colonoscopy, sigmoidoscopy with stool haemoglobin, or computed tomographic colonoscopy, and (8) mammography or breast sonography in women and/or thyroid sonography. Controls with confirmed cancer, suspected cancer, or inflammatory conditions that needed medical management were excluded, resulting in 171 control samples. In the early morning before medical treatment or anaesthesia, all blood samples were collected from fasting participants. Peripheral blood was collected using 5 ml syringes and stored in SST II tubes (Becton Dickinson, Franklin Lakes, NJ, USA) at room temperature for 1 h. Samples were centrifuged at 3000 **g** for 5 min. Supernatants were collected and stored at −80°C. All participants gave informed consent. This study was approved by the Institutional Review Board at Seoul National University Hospital (H-0910-068-298).

Clinicopathological data on demographics and tumour characteristics (stage and size) were available for each patient whose serum was used for this study. Patients were divided into four groups on the basis of age (<49, 50–59, 60–69, and >70-year-old). The T status, N status, and TNM stage of each tumour were classified according to the 6th edition of the AJCC classification (Sobin and Wittekind, 2002). Tumour sizes were divided into two groups (⩽2 and >2 cm) according to the size limit for endoscopic submucosal dissection (Gotoda, 2007).

Serum samples from 120 patients with gastric adenocarcinoma (52 from period 1 and 68 from period 2) and 120 control serum samples were grouped as a training/test set (set 1) for the identification of the diagnostic panels. Serum samples from 95 patients with gastric adenocarcinoma (40 from period 1 and 55 from period 2) and 51 control serum samples were grouped as the independent validation set (set 2). Patient demographics and clinical profiles are presented in Table 1.

### Construction of 29-plex array platform and biomarker assay

For profiling gastric cancer-specific signatures using small samples, an antibody-based bead array method was used. Antibody-based microarray is one of the data-driven approaches, which bypass the identification step for individual markers, making this a faster and more direct method for profiling protein expression and translating this information (Kim *et al*, 2009a). And the xMAP bead-based technology (Luminex Corp., Austin, TX, USA) permits simultaneous analysis of numerous analytes in a single sample. This was successfully applied to identify serum profiles predicting responses to neoadjuvant chemotherapy in locally advanced breast cancer (Nolen *et al*, 2008). Recently, the characteristic serum profiles associated with breast cancer was reported using an antibody-based bead array platform (Kim *et al*, 2009a). For this study, a 29-plex array platform was constructed through an extensive screening process, using a serum bank that had 4500 samples from patients with cancers of the breast, colon, stomach, liver, and lung, as previously described (Kim *et al*, 2009a). From the same serum bank, haptoglobin *α*, transthyretin (TTR), Apolipoprotein A4, and Pro-apolipoprotein A1 (proApoA1) were identified through 2D-PAGE. *β*2-microglobulin, α-1-antitrypin, C-reactive protein, haemoglobin, apolipoprotein A2, apolipoprotein C3, and vitamin D-binding protein were identified through SELDI-TOF MS. On the basis of searching journals, another 18 serum proteins known to be cancer-associated were selected. Sandwich enzyme-linked immunosorbent assays for individual markers were used for validation, resulting in 29 candidates markers (Table 2). This 29-plex array platform was consisted of 4 multiplex kits and 18 simplex kits. A total of 16 markers were classified as four groups according to dilution factor and absence of cross reactivity, and were analysed through four multiplex kit. The remaining 18 markers were analysed through individual simplex kits. Multiplex assays were performed using the xMAP bead-based technology (Luminex). Simplex assays were conducted using a conventional ELISA method, commercially available ELISA kits according to manufacturers’ instructions, a fully automated system for plasma protein determinations (BN II System; Siemens Healthcare Diagnostics, Marburg, Germany), or a bead array method. Bead array kits or antibodies for the construction of the 29-plex panel were purchased as listed in Table 2. Using the platform, the assays for each marker in set 1 were conducted simultaneously, and those in the set 2 were conducted simultaneously.

### Data analysis

For identification of diagnostic algorithms based on the biomarker panel, two ratios of markers (ApoA1/proApoA1and fPSA/tPSA) were additionally analysed. Prior to multivariate classification analyses, values were transformed into log value. Initially, the differences of serum biomarkers between two periods and between control group with normal and abnormal endoscopic findings were analysed. A receiver operating characteristic curve was constructed, and the area under the curve was calculated. As 29 markers were too many for one algorithm, the feature selection process, which reduced biomarkers included in one algorithm, was performed using random forests (RF). The average importance set at 100 was calculated using RF in set 1 (Breiman, 2001). A total of 13 ranked markers were selected for multivariate classification analysis.

For identification of algorithms distinguishing controls from patients with gastric carcinoma, two classification analysis methods, RF and support vector machine (SVM), were used. Random forest, proposed by Berinman, is a combination of tree predictors such that each tree depends on the values of a random vector sampled independently and with the same distribution for all trees in the forest (Breiman, 2001), and the idea behind SVMs is construction of a seperating hyperplane or set of hyperplanes in a high or infinite dimensional space for classification (Vapnik, 1995; Furey *et al*, 2000; Dossat *et al*, 2007). This seperating hyperplane is optimal in the sense of being a maximal distance to the nearest training data points of any class. Among the 240 samples in set 1, 70 samples from patients with gastric adenocarcinoma and 70 control samples were randomly assigned to training sets. The remaining 50 samples were assigned to test sets. RF and SVM were used with the training set to classify individuals as patients with gastric adenocarcinoma or controls. After training, each classification algorithm with different sets of classifiers was cross-validated with the test set. The prediction performances with accuracy (number of patients or controls identical to the result of the classification/examined total number), sensitivity (number of patients classified as patient with gastric adenocarcinoma/number of patients with gastric adenocarcinoma who were examined), and specificity (number of controls classified as control/number of controls who were examined) obtained from 50 randomly partitioned data sets were analysed. Four classification algorithms with selected biomarkers obtained from the experiment with set 1 were tested, without knowledge of true diagnosis, on an independent validation set (set 2). An ROC curve was constructed and the area under the curve was calculated. A *P*-value of <0.05 was considered statistically significant. The R program package (The R project of statistical computing, Wirtschaftsuniversität, Wien, Austria) was used for classification analysis to develop the algorithms.

## Results

**Figure 1:**
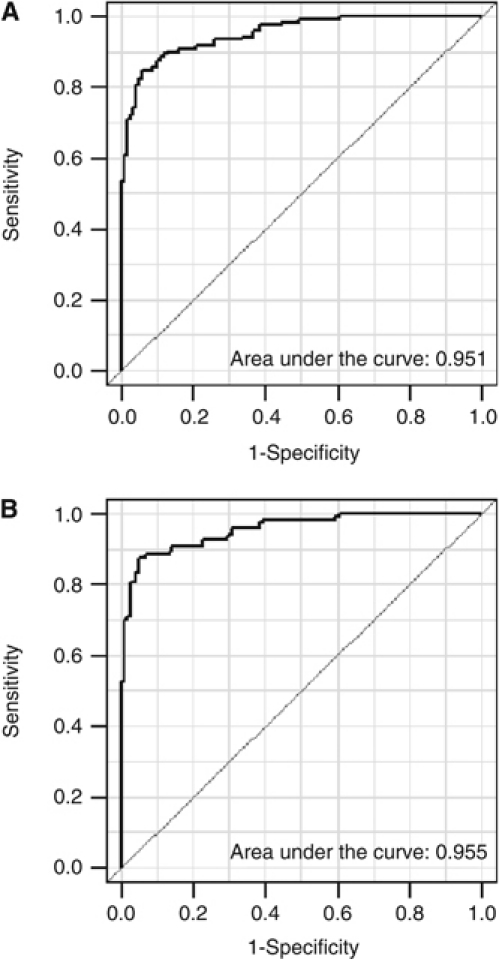
Receiver operating characteristic (ROC) curve and the area under the curve according to two algorithms by classification analysis methods. (**A**) ROC curve of the algorithm by random forest. (**B**) ROC curve of the algorithm by support vector machine.

There were no different levels of biomarkers between two periods and between controls with normal or abnormal endoscopic findings (data not shown). Via RF on set 1, 13 markers as Table 3 were selected for multivariate classification analysis. To identify biomarkers that distinguished sera from patients with gastric adenocarcinoma from control sera, multivariate classification analysis with RF, and SVM was performed on the training set. The training set consisted of 70 serum samples from gastric adenocarcinoma patients and 70 control samples from set 1. After training, each algorithm with a different biomarker panel was cross-validated on the test set, to which the remaining 50 samples were assigned. The accuracy and classification error for two algorithms were calculated for each training and test set. The classification algorithms with the top five ranked average accuracies are summarised in Table 4. The RT and SVM algorithms with the highest average accuracies distinguished between gastric adenocarcinoma and controls, with mean accuracies of 88.3% and 89.7%, respectively. These two algorithms were further tested on a separate, independent blinded set of 95 gastric adenocarcinoma sera and 51 control sera. The mean accuracies of the algorithms determined by RF and SVM were 89.2% and 85.6%, respectively. These accuracies were similar to those from set 1. Among the two algorithms, the biomarker panel from the RF algorithm containing 11 markers showed a higher accuracy in the validation set, though the area under the curve values of two algorithms were similar (Figure 1).

When analysing the diagnostic sensitivity according to TNM stage and tumour size, the sensitivity of the algorithms did not vary much from the overall sensitivity. However, the sensitivity for detecting the early-stage disease was slightly lower than that for advanced disease. Likewise, the sensitivity for tumours with sizes of ⩽2 cm was slightly lower than that for tumours of >2 cm (Table 5). RF algorithms generally outperformed the SVM algorithms, regardless of TNM stage or tumour size. Additionally, SVM algorithms showed a higher sensitivity for small tumours.

## Discussion

Although gastroscopy is the most reliable diagnostic method for detecting gastric adenocarcinoma, it is less accurate at detecting benign gastric diseases, such as peptic ulcers, especially in areas with low-to-intermediate rates of gastric cancer. The average percentage of missed diagnoses by endoscopy was reported to be 0.46–14%, but as high as 33%, depending on the country (Bramble *et al*, 2000; Amin *et al*, 2002; Yalamarthi *et al*, 2004; Voutilainen and Juhola, 2005; Raftopoulos *et al*, 2010). This variation might have been caused by inter-observer variation, suboptimal correlations with histopathology, unimpeded visualization of all anatomic sub-regions, such as remnant stomach after surgery, and delays in receiving endoscopy after anti-secretory medications (Bramble *et al*, 2000; Voutilainen and Juhola, 2005). Moreover, possible discomfort and anxiety of patients who underwent endoscopy led to prevalent use of conscious sedation, which caused adverse outcomes of gastroscopy with an incidence of 0.54% and fatalities of 0.03% (Froehlich *et al*, 1995; Abraham *et al*, 2002).

To overcome these limitations and invasiveness of gastroscopy, we developed a novel panel of biomarkers that differentiate between patients with gastric adenocarcinoma and healthy controls. During development, cancer cells secrete proteins required for tumour growth within the microenvironment of the incipient tumour. Additionally, cancer systemically mounts an immunological defense, which consists of innate and adaptive responses, including autoantibody production and migration of inflammatory cells such as macrophages, histiocytes, and lymphocytes into the tumour (Ohno *et al*, 2003; Patz *et al*, 2007; Yuan *et al*, 2008). As gastric adenocarcinoma is considered to have heterogeneous phenotypes, we predicted that a combination of tumour-expressed and host-response proteins would yield candidate biomarkers.

Two algorithms determined by RF, and SVM analysis differentiated between serum samples from gastric adenocarcinoma patients and control serum in the training/test set with high accuracy (>88%). These algorithms also accurately classified in the validation set (>85%). The sensitivity for accurately diagnosing advanced-stage and large tumours was slightly better than that for diagnosing early-stage and small tumours. However, the overall sensitivity was sustained regardless of TNM stage and tumour size. Although none of the individual markers showed sufficient diagnostic power independently, the biomarker panel identified in this study performed well. The RF algorithm containing 11 biomarkers outperformed the other algorithms, regardless of TNM stage or tumour size. However, the SVM algorithm performed well for diagnosing small tumours.

Several reports have successfully profiled serum proteins for the diagnosis of gastric cancer (Liang *et al*, 2006; Ren *et al*, 2006; Lim *et al*, 2007). These studies emphasised the feasibility of proteomics methodologies rather than biomarkers, and adopted only three to four markers as classifiers after profiling was performed on a small number of cases. In this study, identification of candidate markers was based on analysis of a serum bank consisting of 4500 serum samples and previously published literature. The development and validation of these biomarker panel algorithms was performed using a considerable number of cases.

The diagnostic biomarker panel algorithms in this study have some weaknesses. First, the number of classifiers in these biomarker panels was higher than other previously reported panels (Patz *et al*, 2007; Ueland *et al*, 2010; Yurkovetsky *et al*, 2010). Two algorithms included more than eight classifiers. Possibly, gastric cancer does not have concrete biomarkers that can distinguish cancer from control serum samples as do breast and ovarian cancers. Additional biomarkers might enhance the diagnostic accuracy; however, more markers mean increased cost and time requirements. Second, except for DD and C-reactive protein, the levels of all algorithm-selected biomarkers were significantly lower in patients with gastric adenocarcinoma than in the controls (data not shown). It is more difficult and less accurate to assay low levels of biomarkers than high levels. The accuracy of the assay for measuring decreased biomarker levels can be related to the method of analysis and the skill of the technician. Therefore, simple and cheap methods for analysis of the biomarkers, such as multiplex-bead arrays, are needed. Third, important biomarkers might not have been identified as classifiers because early-stage tumours were included much more often than were advanced-stage tumours in this study.

## Conclusions

Two diagnostic biomarker panel algorithms that included eight to eleven biomarkers, including epidermal growth factor receptor (EGFR), TTR, ApoA1/proApoA1, proApoA1, RANTES, ApoA1, DD, vitronectin, IL-6, C-reactive protein, A2M, and PAI-1, were developed and validated. Among the diagnostic algorithms, the RF algorithm with more classifiers than the others, in general, outperformed the others regardless of TNM stage or tumour size. The SVM algorithm performed well for the diagnosis of small tumours. These two less-invasive biomarker panels could supplement clinical gastroscopic evaluation of symptomatic patients to enhance diagnostic accuracy. Further studies in more patients are required to refine the diagnostic algorithms with the expectation that an efficient, optimal diagnostic strategy will improve patient outcomes.

## Figures and Tables

**Table 1:** Patient's demographics and clinical profiles

	**Training/test set (set1)**		**Validation set (set2)**	
	**Gastric adenocarcinoma (*n*=120)**	**Control (*n*=120)**	**Gastric adenocarcinoma (*n*=95)**	**Control (*n*=51)**
Age, years	60.8±10.4	52.0±6.0	59.4±11.1	52.1±6.6
				
*Sex*				
Male	75	66	59	28
Female	45	54	36	23
				
*Period of sample*				
Period 1 (Nov. 2002–Dec. 2003)	52		40	
Period 2 (Jul. 2006–Aug. 2007)	68		55	
				
*Stage*				
I	69		59	
II	25		16	
III	17		12	
IV	9		8	
				
*Endoscopic findings*				
No abnormality		19		8
Gastritis or ulcer		98		40
Hyperplastic/fundic polyp		1		3
Others		2		0

**Table 2:** List of 29 markers

**Biomarkers**	**Antigen**	**Primary antibody**	**Secondary antibody**	**Kit**
*Oncofetal protein*				
AFP^a^				Rules-based medicine (Austin, TX, USA)
CA 125^a^				Rules-based medicine
CA 19-9 a				Rules-based medicine
CEA^a^				Rules-based medicine
fPSA^a^				Rules-based medicine
PSA^a^				Rules-based medicine
*Tumour suppressor protein*				
DKK.3^a^				R&D Systems (Minneapolis, MN, USA)
				
*Cytokine/chemokine*				
IL-6^a^				Millipore (St Charles, MO, USA)
RANTES^a^				Millipore
				
*Immune/inflammation*				
*β* 2M^b^				Siemens Healthcare Diagnostics (Marburg, Germany)
				
*Factor/hormone*				
EGFR^a^				R&D Systems
				
*Acute phase protein*				
A1AT^b^	Sigma-Aldrich (St Louis, MO, USA)	Acris Antibodies (Hiddenhausen, Germany)	Biodesign International (Saco, ME, USA)	
A2M^a^	EMD Chemicals Inc. (San Diego, CA, USA)	R&D Systems	Affinity Bioreagents, Inc. (Golden, CO, USA)	
CRP^b^				Siemens Healthcare Diagnostics
DD^a^	Abcam (Cambridge, UK)	Biodesign International	Biodesign International	
Hp^c^				Siemens Healthcare Diagnostics
TTR^c^				Siemens Healthcare Diagnostics
				
*Coagulation/thrombosis*				
Hg^b^	Sigma-Aldrich	Biodesign International	Bethyl (Montgomery, TX, USA)	
PAI-1^a^	EMD Chemicals Inc.	Abcam	US Biological (Swampscott, MA, USA)	
				
*Metabolism*				
ApoA1^a^				Siemens Healthcare Diagnostics
ApoA2^a^				Millipore
ApoA4^c^	Bioinfra (Seoul, Korea)	Santa Cruz (Santa Cruz, CA, USA)	AbFrontier (Seoul, Korea)	
ApoC2^b^				Millipore
ApoC3^b^				Millipore
proApoA1^c^	Bioinfra	Biodesign International	Biodesign International	
				
*Adhesion*				
sICAM-1^a^				Millipore
sVCAM-1^a^				Millipore
VN^a^	Biodesign International	Biodesign International	Chemicon (Temecula, CA, USA)	
				
*Others*				
VDBP^b^	Biodesign International	Abcam	Abcam	

Abbreviations: AFP=α-fetoprotein; Apo=apolipoprotein; A1AT=α-1-antitrypin; A2M=α-2 macroglobulin; *β* 2M=*β*2-microglobulin; CA=cancer antigen; CEA=carcinoembryonic antigen; CRP=C-reactive protein; DD=D-dimer; DKK.3=Dickkopf 3; EGFR=epidermal growth factor receptor; fPSA=free prostate-specific antigen; Hg=haemoglobin; Hp=Haptoglobin *α*; IL=interleukin; PAI-1=plasminogen activator inhibitor-1; proApo=pro-apolipoprotein; PSA=prostate-specific antigen; RANTES=regulated upon activation, normally T-expressed and presumably secreted; sICAM=soluble intercellular cell adhesion molecule-1; sVCAM=soluble vascular cell adhesion molecule-1; TTR=transthyretin; VDBP=vitamin D-binding protein; VN=vitronectin.

a Twenty three markers were selected through literature search.

b Seven markers were discovered through surface-enhanced laser desorption/ionization time-of-flight (SELDI-TOF) mass spectrometry. A1AT, *β* 2M, Hg, and VDBP were identified from the peaks, which were differently represented in especially gastric adenocarcinoma serum compared with control serum.

c Four markers were discovered through two-dimensional polycarylamide gel electrophoresis (2D-PAGE) using the serum of patients with gastric adenocarcinoma.

**Table 3:** Biomarker selection in set 1

**Marker**	**Avg.Imp**	**Rank**	***P*-value**
EGFR	30.13	1	0.000
ApoA1/proApoA1	29.70	2	0.000
TTR	29.17	3	0.000
proApoA1	26.89	4	0.000
RANTES	26.78	5	0.000
ApoA1	25.35	6	0.000
ApoA2	24.60	7	0.000
DD	24.03	8	0.000
VN	23.61	9	0.000
IL-6	18.71	10	0.000
CRP	18.47	11	0.000
A2M	17.25	12	0.000
PAI-1	16.80	13	0.000
			
ApoC3	16.31	14	0.000
ApoC2	15.24	15	0.000
fPSA	14.08	16	0.118
VDBP	13.24	17	0.000
AFP	12.98	18	0.004
Hp	11.73	19	0.043
sVCAM-1	11.37	20	0.000
DKK3	10.94	21	0.000
ApoA4	9.45	22	0.309
CA19-9	9.13	23	0.525
A1AT	8.99	24	0.349
Hemo	8.96	25	0.663
PSA	8.24	26	0.404
sICAM-1	7.77	27	0.702
fPSA/tPSA	7.16	28	0.875
CA125	6.84	29	0.401
CEA	6.29	30	0.877
B2M	5.79	31	0.962

Abbreviations: AFP=α-fetoprotein; Apo=apolipoprotein; A1AT=α-1-antitrypin; A2M=α-2 macroglobulin; *β* 2M=*β*2-microglobulin; CA=cancer antigen; CEA=carcinoembryonic antigen; CRP=C-reactive protein; DD=D-dimer; DKK.3=Dickkopf 3; EGFR=epidermal growth factor receptor; fPSA=free prostate-specific antigen; Hp=Haptoglobin *α*; IL=interleukin; PAI-1=plasminogen activator inhibitor-1; proApo=pro-apolipoprotein; PSA=prostate-specific antigen; RANTES=regulated upon activation, normally T-expressed and presumably secreted; sICAM=soluble intercellular cell adhesion molecule-1; sVCAM=soluble vascular cell adhesion molecule-1; tPSA=total prostate-specific antigen; TTR=transthyretin; VDBP=vitamin D-binding protein; VN=vitronectin.

**Table 4:** Diagnostic performance of classification algorithms with biomarker panel

		**Training/test set (set1)**			**Validation set (set2)**		
**Marker combination**	**Number of markers**	**Accuracy (%)**	**Sensitivity (%)**	**Specificity (%)**	**Accuracy (%)**	**Sensitivity (%)**	**Specificity (%)**
*RF classification analysis*							
EGFR, TTR, proApoA1, RANTES, ApoA1, DD, VN, IL-6, CRP, A2M, PAI-1	11	88.3	90.1	86.4	89.2	88.8	89.7
EGFR, TTR, proApoA1, RANTES, ApoA1, DD, VN, IL-6, A2M, PAI-1	10	88.0	89.3	86.7			
EGFR, TTR, proApoA1, RANTES, ApoA1, DD, VN, IL-6, CRP, PAI-1	10	87.9	89.2	86.6			
EGFR, proApoA1, TTR, proApoA1, RANTES, DD, VN, IL-6, CRP, A2M, PAI-1	11	87.9	89.1	86.7			
EGFR, proApoA1, TTR, proApoA1, RANTES, ApoA1, DD, VN, IL-6, CRP, A2M, PAI-1	12	87.8	89.2	86.4			
							
*SVM classification analysis*							
EGFR, ApoA1/proApoA1, TTR, RANTES, DD, VN, IL-6, A2M	8	89.7	91.8	87.7	85.6	89.7	81.6
EGFR, ApoA1/proApoA1, TTR, RANTES, ApoA1, DD, VN, IL-6, A2M	9	89.2	91.2	87.2			
EGFR, ApoA1/proApoA1, TTR, RANTES, DD, VN, IL-6, CRP, A2M, PAI-1	10	89.1	90.8	87.5			
EGFR, ApoA1/proApoA1, TTR, RANTES, ApoA1,DD, VN, IL-6, CRP, A2M, PAI-1	11	89.1	91.1	87.1			
EGFR, ApoA1/proApoA1, TTR, RANTES, DD, VN, IL-6, A2M, PAI-1	9	89.1	90.6	87.5			

Abbreviations: Apo=apolipoprotein; A2M=α-2 macroglobulin; CRP=C-reactive protein; DD=D-dimer; EGFR=epidermal growth factor receptor; IL=interleukin; PAI-1=plasminogen activator inhibitor-1; proApo=pro-apolipoprotein; RANTES=regulated upon activation, normally T-expressed and presumably secreted; TTR=transthyretin; VN=vitronectin.

**Table 5:** Comparison of sensitivity according to size and TNM stage

		**Sensitivity (%) according to size**		**Sensitivity (%) according to TNM stage**	
**Algorithm**	**Set**	**⩽2 cm**	**>2 cm**	**Stage I–II**	**Stage III–IV**
RF	Training/test set	87.0	92.6	91.0	94.4
	Validation set	81.8	95.7	92.3	92.9
					
SVM	Training/test set	91.3	91.6	90.0	94.4
	Validation set	72.7	94.3	88.5	92.9

Abbreviations: RF=random forests; SVM=support vector machine.
